# Effect of closed vacuum aspiration technique on lung collapse time in thoracoscopic anatomical segmentectomy

**DOI:** 10.1097/MD.0000000000015447

**Published:** 2019-05-24

**Authors:** Zhenyang Zhang, Chuangcai Yang, Jiangbo Lin, Junjie Hong, Yunyang Zhuang, Mingqiang Kang

**Affiliations:** a Department of Thoracic Surgery, Fujian Medical University Union Hospital; b Key Laboratory of Ministry of Education for Gastrointestinal Cancer; c Fujian Key Laboratory of Tumor Microbiology, Fujian Medical University, Fuzhou, China.

**Keywords:** lung expansion and collapse method, thoracoscopic anatomical segmentectomy, vacuum aspiration technique

## Abstract

To investigate the effect of lung expansion and collapse method combined with closed vacuum aspiration technique on lung collapse time, reduce the waiting time of surgery.

Forty patients with pulmonary peripheral nodules under thoracoscopic anatomical segmentectomy were divided into 20 cases of natural collapse group and 20 cases of modified collapse group. The natural collapse group used the traditional natural collapse method, and the modified collapse group used a lung expansion and collapse method combined with closed vacuum aspiration technique to record the lung collapse time and compare them.

Thoracoscopic anatomical segmentectomy was successfully performed in both groups. The lung collapse time in the natural collapse group and the modified collapse group was 17.08 ± 1.35, 8.90 ± 0.39, respectively, *P* < .05.

The lung expansion and collapse method combined with closed vacuum aspiration technique can reduced the waiting time of lung collapse during thoracoscopic anatomical segmentectomy, and can processed the inter-segment boundary better, thereby reduced the waiting time of surgery.

## 1 Introduction

Peripheral pulmonary nodules refer to lesions located in the 1/3 lateral zone of the lung with a diameter of less than 2 cm and a solid component greater than 50%. These nodules may be inflammatory masses in the lungs, benign, or early stage lung cancer.^[1,2]^ In recent years, with the rapid development of chest imaging, the detection rate of peripheral pulmonary nodule lesions has increased significantly.^[3]^ For peripheral pulmonary nodules, the main treatment of thoracotomy in the past, but the short incision, the large amount of intraoperative blood loss, the patient's physical quality requirements, so that its clinical application is limited.^[4]^With the continuous development of minimally invasive techniques, thoracoscopic techniques are becoming more and more mature, providing a new way for the diagnosis and treatment of peripheral pulmonary nodules.

In order to preserve lung function as much as possible, thoracoscopic anatomical segmentectomy is more preferred for surgical selection of peripheral pulmonary nodules.^[5,6]^Thoracoscopic anatomical segmentectomy has proven to be an effective treatment for this nodule. In order to achieve anatomical resection and give full play to the function of retaining the lung segment, it is necessary to accurately remove the target segment bronchus and arteries, preserve the inter-segment vein, and determine the inter-segment boundary through lung collapse.^[7]^ The 3 difficult points of lung segment resection are the location of the pulmonary nodule, the dissection of the artry/vein/bronchus of the target segment, and the judgment of the inter-segment boundary. The judgment of the inter-segment boundary is particularly difficult because it is not visible. At present, there are methods such as inflation method, fluorescent staining method, and expansion collapse method.

The lung expansion and collapse method is now widely used in clinical practice, and it can be found in clinical practice that it takes a long time to collapse after the lung expands, which extends surgery time. Vacuum aspiration technique has been widely used in various thoracic surgery in clinical practice.^[8]^ Therefore, this experiment uses closed vacuum aspiration technique to study its effect on lung collapse time.

## 2 Materials and methods

### 2.1 Clinical data

A retrospective analysis of 40 patients with peripheral pulmonary nodules under thoracoscopic anatomical segmentectomy from March 2016 to May 2017 at the Fujian Medical University Union Hospital. Inclusion criteria: the solid nodules of the patients were less than 2 cm in diameter, and there was no metastasis of other organs such as brain, bone, kidney; cardiopulmonary function can tolerate general anesthesia; other preoperative examinations such as blood, urine, electrocardiogram, chest lateral position, and CT (computed tomography) examination showed no obvious surgical contraindications. Exclusion criteria: multiple pulmonary nodules and diameter greater than 2 cm; imaging evidence of other organ metastasis; poor cardiopulmonary function; severe infection in other parts of the body.

Forty patients who met the inclusion criteria were divided into 20 cases of natural collapse group and 20 cases of modified collapse group according to the traditional natural collapse method and lung expansion collapse method combined with closed vacuum aspiration technique, respectively.

### 2.2 Methods

Preoperative preparation: 3D-CTBA (3-dimensional computed tomography bronchography and angiography) was used to establish a 3-dimensional model based on thin-layer CT imaging data. The reconstruction software mimics19.0 was used to reconstruct 3D images of lung bronchus, blood vessels and lung nodules, and the surgical approach was planned.

**Figure 1:**
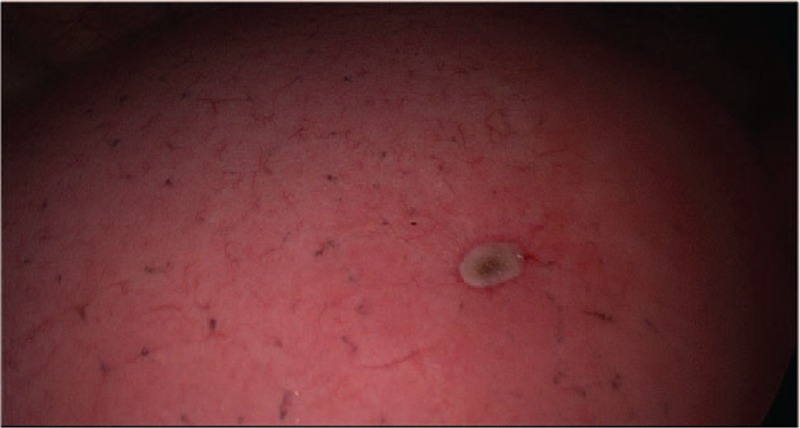
Swell the affected lung to pinkwith pure oxygen, and start timing.

**Figure 2:**
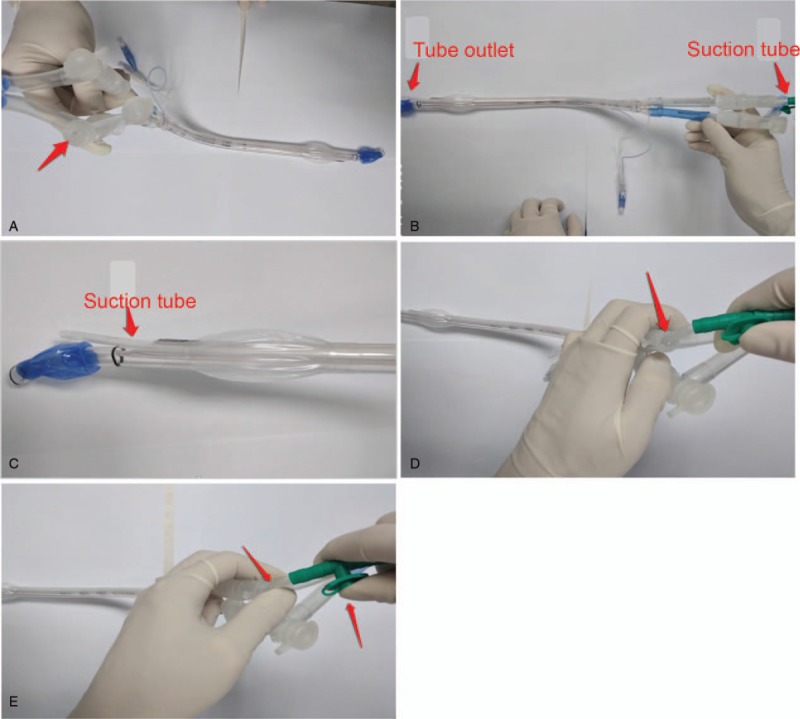
Two groups of lung collapse method steps. (A) Natural collapse method. Modified collapse group:(B) Suction tube is inserted by the right nozzle; (C) Suction tube out of the right tube; (D) Open the right nozzle cover, there is a gap between the suction tube and the right tube; (E) Squeeze the closed gap with your fingers and adjust the negative pressure suction with the other hand.

Surgery and anesthesia: All patients underwent general anesthesia with double-lumen endotracheal intubation, healthy side position, and healthy side lung ventilation. The classic laparoscopic three-hole method was selected. According to the 3D model of preoperative reconstruction, after cutting off the target segment of bronchus and arteries, double lung ventilation (airway pressure maintained at 20 cm H_2_O), swell the affected lung to pinkwith pure oxygen (Fig. 1), and start timing. For the patients in the natural collapse group, the affected side tube of the double lumen endotracheal tube was communicated with the outside, and the affected side lung collapsed naturally (Fig. 2A). For patients in modified collapse group, a common suction tube (type I 4.0 mm (F12) × 480 mm) was completely inserted into the affected side tube of the double lumen endotracheal tube, which was pierced from the bottom and externally closed vacuum suctioned (40.0–53.3 Kpa) and manually operated to promote the lung collapse of the affected side (Fig. 2B–E).

**Figure 3:**
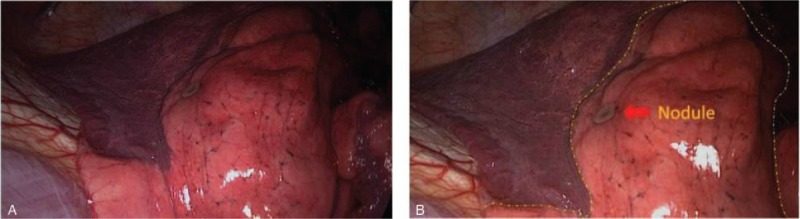
Standard clear boundary line between the target segment lung and other lung segments, stop timing, and record the lung collapse time.

A clear boundary line (Fig. 3A and B) between the target segment lung and other lung segments, stop timing, and record the lung collapse time.

### 2.3 Statistical analysis

All data were analyzed by SPSS20.0 statistical software. The measurement data were described by 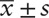 and the *t* test was used for statistical analysis. The difference was statistically significant at *P* < .05.

## 3 Results

### 3.1 Baseline situation

The patient's basic information, clinical manifestations, and diseased region were shown in Table 1. The differences were not statistically significant (*P* > .05).

**Table 1:**
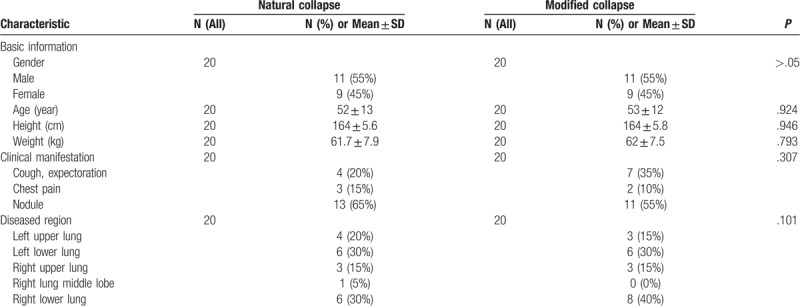
Baseline characteristics of the patient.

### 3.2 Lung collapse time

Both groups of patients successfully completed thoracoscopic anatomical segmentectomy. All patients had no complications such as hemoptysis, air leak, and pulmonary edema. The pathology was benign in 2 cases, metastatic carcinoma in 3 cases, and primary lung cancer in 35 cases. The lung collapse time in the natural collapse group and the modified collapse group was 17.08 ± 1.35, 8.90 ± 0.39, respectively, *P* < .05 (Table 2).

**Table 2:**
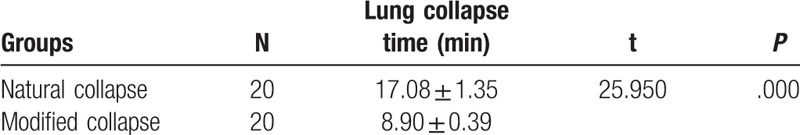
Comparison of lung collapse time (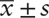).

## 4 Discussion

In the current study, we illustrated the effect of closed vacuum aspiration techniques on lung collapse time. We found that the lung expansion and collapse method combined with closed vacuum aspiration technology significantly reduced lung collapse time and reduced operation time. So far, there has not been a study on the combination of closed vacuum aspiration techniques to reduce the time of lung collapse. Therefore, the results of this study are of great significance for better treatment of the inter-segment boundary and completion of thoracoscopic anatomical segmentectomy.

With the application and popularization of CT and other imaging examinations, the diagnostic rate of peripheral pulmonary nodules has increased, especially the detection rate of lung nodules less than 2 cm in diameter has been significantly improved.^[9,10]^ With the continuous development of minimally invasive techniques, thoracoscopic anatomical segmentectomy is increasingly used for the diagnosis and treatment of peripheral pulmonary nodules.^[11,12]^At present, in order to achieve accurate thoracoscopic anatomical segmentectomy, to ensure the lung function of patients after lung surgery, the combination of preoperative 3D-CTBA, intraoperative interfacial plane boundary defined by lung expansion and collapse method, and the segment of the lung retained after resection of the target segment of the lung can be close to the original geometry after expansion.^[13]^

A major difficulty in segmental resection is the determination of the inter-segment boundary. At present, the methods for determining the inter-segment boundary are inflation method, fluorescent staining method, and expansion collapse method.^[14]^The inflation method is related to the dispersion of the lung. If there is secretion in the airway, the inter-segment boundary is easily inaccurate, and the inflation gas is too large, causing some non-target segment tissue to inflate, resulting in miscut. Fluorescence staining shows that the inter-segment boundary has a short duration, requires special equipment, and the deep tissue interface cannot be displayed clearly. The modified expansion collapse method does not require special equipment, and shows that the inter-segment boundary lasts for a long time, which can guide the next step of opening the lung segment and ensure the safe margin of the tumor. Therefore, in the clinical aspect, the inter-segment boundar is judged mainly according to the expansion collapse method.^[15–17]^

In this experiment, after cutting off the segmental bronchus, we used the lung expansion and collapse method to determine the inter-segment boundary. The lung expansion and collapse method is that the blood flow takes away the oxygen in the healthy lung segment and causes collapse, at the same time, the lung tissue of the target segment cannot be collapsed because the blood flow is blocked, thereby causing the inter-segment boundary. At present, in the clinical period, the time of intraoperative lung expansion and retraction to collapse is about 15 ± 3 minutes. In order to reduce this waiting time, we inserted a common suction tube into the affected side tube of the double lumen endotracheal tube and promoted the collapse after re-expansion combining with closed vacuum aspiration technology. Closed vacuum aspiration techniques can accelerate the collapse of healthy lungs, while the target segment of the lung trachea has been cut, the cohn hole is a flap, and the gas in the target segment of the lung will not be sucked out.^[18,19]^ This allows for faster determination of the inter-segment boundary and reduces the waiting time for surgery. Similarly, our results showed the lung collapse time of the lung expansion and collapse method combined with vacuum aspiration technique was significantly lower than that of the natural collapse group (*P* < .05). The modfied collapse inter-segment boundary (Fig. 3B) using closed vacuum aspiration techniques was confirmed to be the same as the naturally- collapsed inter-segment boundary (Fig. 3A) in 5 patients who accepted the two methods during the surgery before this experiment. There were also no obvious complications such as hemoptysis and pulmonary edema in the modified group.

In summary, the lung expansion and collapse method combined with closed vacuum aspiration technology can significantly reduced lung collapse time and reduced the waiting time of operation. Our study provided a certain experimental and theoretical basis for the reduction of lung collapse time and the better treatment of the inter-segment boundary during thoracoscopic anatomical segmentectomy.

## Author contributions

**Conceptualization:** Zhenyang Zhang, Jiangbo Lin, Mingqiang Kang.

**Funding acquisition:** Jiangbo Lin.

**Investigation:** Chuangcai Yang, Yunyang Zhuang, Lei Gao, Jiafu Zhu.

**Software:** Junjie Hong.

**Writing – original draft:** Zhenyang Zhang.

**Writing – review & editing:** Zhenyang Zhang.
